# Investigation and comparison of the influence of modified DBR and yellow color filters for quantum dot color conversion-based micro LED applications

**DOI:** 10.1016/j.heliyon.2024.e35492

**Published:** 2024-08-03

**Authors:** Bao-Le Dai, Jing-Wei Ji, Bing-Han Wu, Kuan-An Chen, Hideki Kuroda, Hung-Chen Kou, Tomohiro Akada, Chun-Yu Li

**Affiliations:** a Department of Electronic Engineering, National Yunlin University of Science and Technology, Douliu, 640301, Taiwan; b Graduate School of Electronic Engineering and Technology, National Yunlin University of Science and Technology, Douliu, 640301, Taiwan; c SynthEdge Advanced Materials Corp. Ltd., Taoyuan, 327008, Taiwan; d Otsuka Tech Electronics Corp. Ltd., Tainan, 700019, Taiwan

**Keywords:** Micro-LED display, Quantum dot, Color conversion, Distributed bragg reflector (DBR), Yellow color filter

## Abstract

This study compares how a modified distributed Bragg reflector (DBR) and yellow color filter (Y-CF) increase the color purity, viewing angle, and brightness of the quantum dot color conversion layer (QDCC) for micro-LED displays. We designed and built a 53-layer high-performance modified DBR with almost total blue leakage filtering (T %: 0.16 %) and very high G/R band transmittance (T %: 96.97 %) for comparison. We also use a Y-CF that filters blue light (T %: 0.84 %) and has good G/R band transmittance (T %: 94.83 %). Due to DBR's angle dependency effect, the modified DBR/QDCC structure offers a remarkable color gamut (117.41 % NTSC) at the forward viewing angle, but this rapidly diminishes beyond 30°. The Y-CF/QDCC structure retains 116 % NTSC color at all viewing angles. Because of its consistent color performance at all viewing angles, sufficient brightness, and outstanding color gamut, the Y-CF/QDCC structure is the best option for contemporary QDCC-based micro-LED displays.

## 1 Introduction

**Fig. 1:**
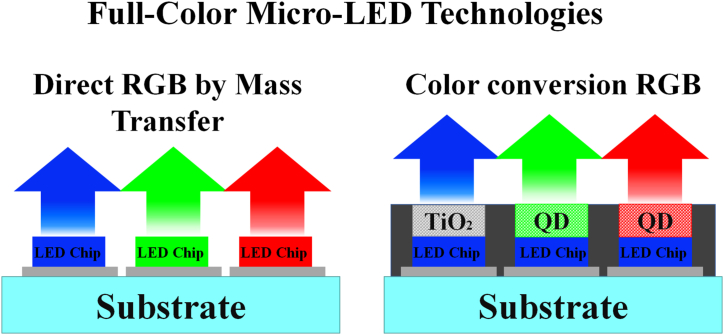
Representation of full-color micro-LED technologies: (a) A matrix comprising of numerous micro-LEDs is arranged directly with red, green and blue micro-LED chips via mass transfer. (b) The RGB sub-pixels are driven by three blue micro-LEDs coated with red and green quantum dots.

Microlight-emitting diodes, or Micro-LEDs, have garnered a lot of interest as potential candidates for next-generation display applications due to their distinctive features, which include their narrow full-width at half-maximum (FWHM), high brightness, low power consumption, and long device lifetime. For many applications, micro-LEDs are a great substitute for traditional liquid crystal displays (LCDs) and organic light-emitting diodes (OLEDs) because of their higher resolution, improved transparency, and faster refresh rates [[1], [2], [3], [4], [5], [6], [7], [8], [9], [10], [11], [12], [13], [14], [15], [16]]. Within the realm of micro-LEDs, it is evident that the chip's dimensions may be directly linked to the pixel per inch (PPI) index, which is a standard indicator of display resolution. Generally speaking, chips smaller than 100 μm will be referred to as micro-LEDs. Currently, mass transfer and color conversion are two distinct techniques that can be employed to achieve a full-color scheme [[17], [18], [19], [20], [21], [22], [23], [24], [25], [26], [27], [28], [29], [30], [31], [32]], as demonstrated in Fig. 1.

Nevertheless, the use of red, green, and blue (RGB) micro-LEDs in the traditional mass transfer method for full-color displays requires precise alignment and is costly. As a result, this presents significant challenges for mass production. Another solution that could potentially ease this issue is implementing a color conversion layer, which would down-convert high-energy photons (blue) into lower-energy ones (red and green) [[33], [34], [35]]. This eliminates the necessity for mass transfer in this method, as the micro-LED arrays may remain monolithically produced, single-color devices. In order to facilitate color conversion, it is essential to use materials with excellent color conversion properties. Several possibilities have been proposed for this purpose, including conjugated polymers, fluorescent dye molecules, conventional phosphors and colloidal quantum dots (CQDs) [[36], [37], [38], [39], [40], [41], [42], [43], [44]]. Over the past few decades, CQDs have attracted considerable interest in academic research.

In general, CQDs as color conversion layers (QDCC) have the potential to enhance color performance, lower costs, and integrate easily with ultra-violet (UV) or blue light micro-LEDs. Compared to conventional phosphors, quantum confinement effects have gained considerable research attention thanks to their exceptional optoelectronic properties that encompass, among others, narrowly adjustable emission wavelengths dependent on size and composition. Additionally, they exhibit excellent color purity, high photoluminescence quantum yield, and impressive longevity. The integration of CQDs with micro-LED displays results in numerous beneficial features. Theoretically, these include high resolution, an extensive range of colors for display, and an exceptional color rendering index for illumination. This amalgamation offers excellent potential for the advancement of display and lighting technologies. For instance, Qun and colleagues deposited red and green quantum dots on a blue LED array to create distinct sub-pixels [45]. Bin et al. attained a color gamut in excess of 83.4 % of the Rec. 2020 color space on the CIE 1931 diagram using micro-LEDs for color conversion [6]. Silvija and colleagues have shown that micro-LEDs converted using QDs have a resolution of over 3600 ppi [46].

However, because the red and green QDCC are not as efficient at absorbing light, lots of blue light is not absorbed and instead mixes with the green and red light when emitted by the green and red QDCC/micro-LEDs. Therefore, the red and green QDCC/micro-LEDs do not have good color quality for display applications [[47], [48], [49]]. As a result, the color gamut of the micro-LED display utilizing color conversion will be diminished.

There are two commonly utilized techniques to improve the color purity of QDCC layers. The first method involves depositing a distributed Bragg reflector (DBR) on the QDCC layer [[50], [51], [52], [53], [54], [55]], while the second method involves applying a color filter (CF) on top of the QDCC layer. When implementing a DBR, it reflects blue light that has not been absorbed and allows green or red light, converted by the QDCC, to pass through. This improves the absorption of blue light by the QDCC, leading to increased efficiency in converting blue light to green or red light, ultimately resulting in a higher intensity of the converted light emission. It is important to note that the reflectivity of a DBR is dependent on the angle of the incident light striking its structure. Hence, if blue light is not directly perpendicular to the DBR, the reflectivity of the structure will be reduced. This is because DBR belongs to an optical structure consisting of multiple transparent thin film layers, which utilizes the principles of interference to achieve specific optical properties. However, as soon as the incident angle deviates from the normal viewing angle, that is, when the light enters obliquely, the optical path difference (or phase difference) changes, resulting in alterations to the optical characteristics compared to the case of normal incidence. In addition, when combining QDCC with color filters, it is typically divided into two stages. Firstly, the red color filter is placed on top of the red QD layer, followed by the green color filter on top of the green QD layer. However, this technique requires different masks for the green and red color filters and epitaxial wafers, increasing costs.

This objective aims to enhance the color saturation, viewing angle, and brightness of the QDCC. In order to accomplish this objective, we conducted a comparative analysis of different viewing angles ranging from 0° to 80°. This analysis is performed using a yellow color filter (referred to as Y-CF) and a modified DBR. Subsequently, we examined the alterations in the emission spectra as a function of the viewing angle. The objective of our research is to provide valuable insights into the most effective methodologies for achieving superior full-color micro-LED displays.

## 2 Experiment

### 2.1 Traditional DBR and modified DBR

Only a few layers of high- and low-refractive-index structures have been studied in DBR micro-LED research at this time. These structures range from 10 to 15 pairs of 21-layer to 31-layer DBR made of TiO_2_/SiO_2_ [[52], [53], [54]]. Nevertheless, this method fails to filter enough blue light. To address this limitation, we employ the optical simulation software of Macleod V12.2 to simulate and optimize a 53-layer modified DBR configuration (Ta_2_O_5_/SiO_2_), aiming to achieve complete blue light filtration. Subsequently, we utilize a thermal evaporator to fabricate the modified DBR, thereby enabling comprehensive blue light filtration. The total thickness of the 53-layer (26 pairs of Ta_2_O_5_/SiO_2_/Ta_2_O_5_) modified DBR film layer is designed to be ∼3.25 μm. The modified DBR design is shown in Supplementary Table S1.

**Fig. 2:**
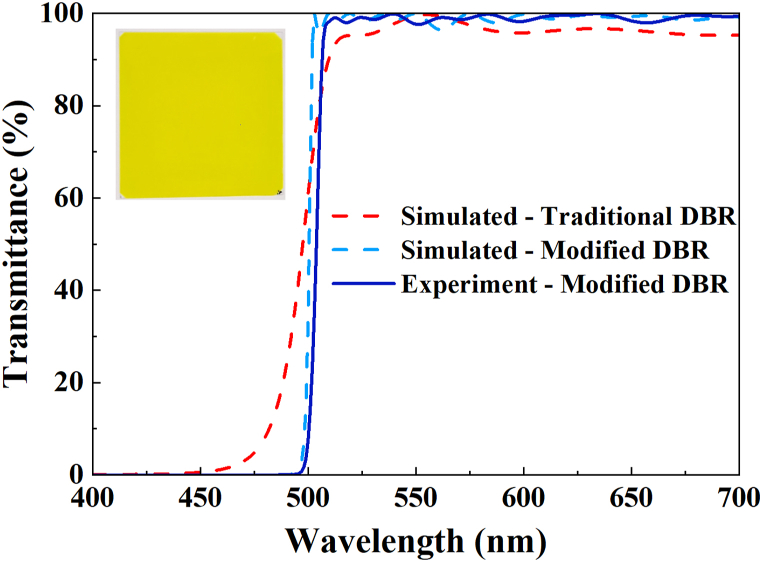
Simulated transmittance spectra of conventional and modified DBR and transmittance spectra of an actual sample of modified DBR‥ (The actual modified DBR coated on a glass substrate is displayed in the upper left.)

Fig. 2 illustrates the transmittance spectra of the simulated and experimental DBR. The average transmittance of the simulated modified DBR, simulated conventional DBR, and modified DBR with an authentic coating on the glass substrate across the wavelength range of 500 nm–700 nm (corresponding to the green and red bands) is found to be 98.47 %, 95.50 %, and 96.97 %, respectively. In addition, the average transmittance within the blue band (400 nm–500 nm) for each is 0.60 %, 6.67 %, and 0.16 %. Furthermore, a subtle distinction exists between the simulated modified DBR and the modified DBR applied to the glass substrate. This issue arises due to the ongoing evaporation process of 53 layers through the thermal evaporator, leading to a certain level of imprecision in the thickness of the film. Nevertheless, our modified DBR coating demonstrates exceptional efficacy in the filtration of blue light. The photograph in the inset displays the modified DBR coated on a glass substrate. The yellow hue observed is a result of a complete reflection of blue.

### 2.2 Yellow, green, and red CF

**Fig. 3:**
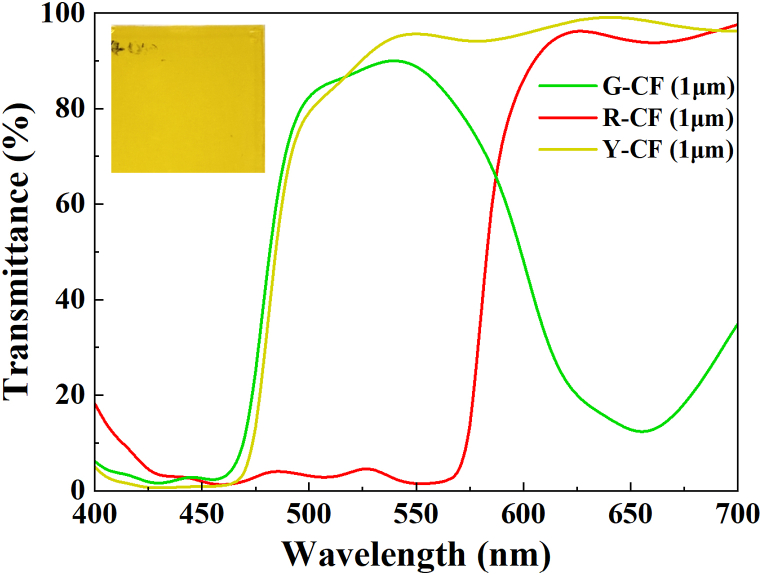
The transmission spectra of yellow, green, and red color filters‥ (Inset in the upper left: the Y-CF, which has a thickness of 1 μm and is coated onto a glass substrate.).

Fig. 3 displays the transmittance spectra of yellow, green, and red CFs, all with a thickness of 1 μm. Pigment CF is used by QDCC to decrease blue light leakage and environmental light interference to improve the color gamut. The figure indicates that Y-CF demonstrates more excellent transmittance in the 500–700 nm range (94.83 %) than G-CF and R–CF and lower transmittance near the blue light wavelength of 446 nm (0.84 %). This suggests that Y-CF is highly effective at filtering blue light energy not absorbed by QDCC and presents improved light emission brightness performance in the R/G band. For this investigation, Y-CF is selected to match with G or R-QDCC to achieve superior light emission brightness and reduced blue leakage compared to matching G-QDCC with G-CF or R-QDCC with R–CF under the excitation of blue light emission. More importantly, Y-CF reduces the number of photolithography steps in the process compared to G-CF and R–CF, thus reducing process costs. The photo in the inset shows Y-CF coated on a glass substrate. The observed yellow hue results from the blue band of white light being absorbed by Y-CF.

### 2.3 Sample preparation

In this research, all the CF and QD solutions are obtained from SynthEdge Advanced Materials Corp. Ltd. To enhance the absorption of blue light for QDCCs, the integration of titanium dioxide (TiO_2_) nanoparticles with an approximate average diameter of 170 nm will be used in conjunction with the solution of QDs. This causes the optical path through the QDCC to be elongated, thereby enhancing the absorbing process. The purpose is mainly to develop high-ppi micro-LED displays. In the case of sub-pixel reduction in area, too thick QDCC (∼10 μm) will hinder the light-emitting viewing angle and reduce the light efficiency and brightness due to the re-absorption effect. Therefore, the thinner the QDCC, the more favorable it is for developing high-ppi micro-LED displays. Consequentially, in QDCC, with a relatively small thickness range of approximately 1.6–3 μm, greater emphasis is placed on optimizing its effectiveness in absorbing blue light and converting light using QDs. The ideal weight ratio between QDs and scattering TiO_2_ nanoparticles in QDCC photoresist is approximately 2:1.

**Fig. 4:**
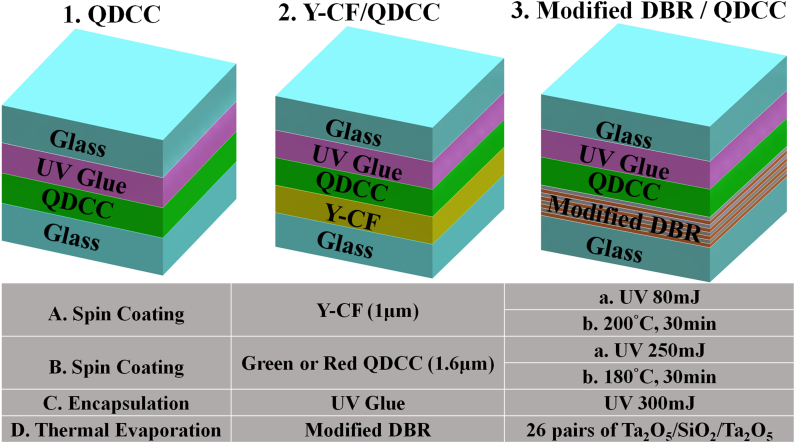
Specific conditions and procedures for the preparation of QDCC, modified DBR/QDCC, and Y-CF/QDCC.

**Fig. 5:**
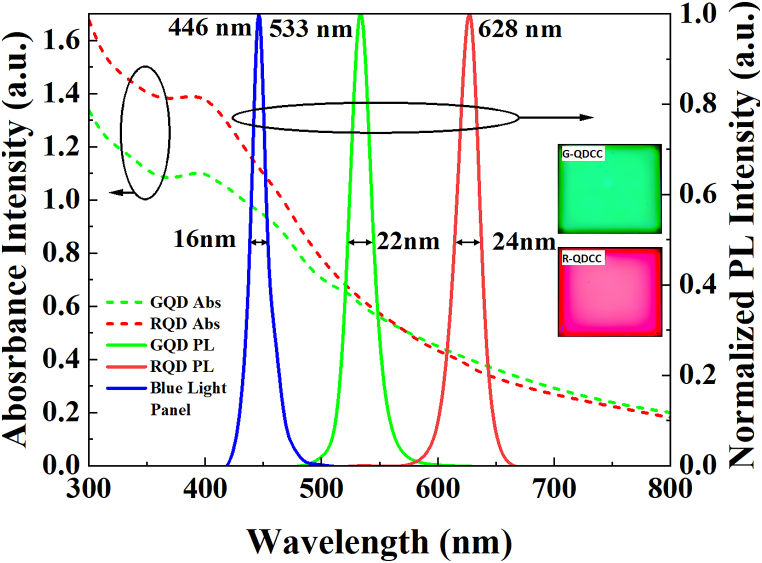
The UV–Visible absorption spectra (shown by dotted lines) and emission spectra (represented by solid lines) of green and red quantum dots that are included into a photoresist containing TiO_2_ nanoparticles in the form of a thin film‥ (Inset on the right: The emissions of light from green QDCC and red QDCC, respectively, are excited by a blue light panel.).

The QDCC/glass and Y-CF/QDCC/glass structures are fabricated on a glass substrate using conventional spin coating, UV curing, and annealing techniques. Subsequently, the sample is encapsulated by applying UV glue to another piece of glass. The above QDCC samples are coated on 10 cm × 10 cm glass with a thickness of 0.3 mm. In addition, the fabrication process of the modified DBR/QDCC involves the sequential application of 26 pairs of Ta_2_O_5_/SiO_2_/Ta_2_O_5_ onto glass using a thermal evaporator to create a DBR structure. Subsequently, QDCC is coated onto the DBR, which is securely sealed with an additional piece of glass employing UV glue. It is essential to mention, however, that to maintain consistency in the modified DBR characteristics of thermal evaporation, we utilize 5 cm × 5 cm glass in this instance. This allows for the simultaneous placement of two pieces of glass into the thermal evaporator, as the maximum effective area for evaporation is 10 cm × 10 cm. Subsequently, these two pieces of modified DBR/glass are applied to G-QDCC and R-QDCC, respectively. Fig. 4 shows the specific parameters and procedures for preparing the QDCC samples. It is important to note that the blue light panel will be located above the QDCC sample in the configuration shown. During the measurement process, the blue light passes through the glass/UV adhesive layer, then through the QDCC, and finally through the Y-CF or modified DBR/glass. UV–vis absorption (dotted lines) and emission (solid lines) spectra of green and red QDCC films on glass, each with a thickness of 1.6 μm, are shown in Fig. 5. This batch of the QDCC is not encapsulated with UV glue in order to avoid any potential interference with the absorbance measurement results. These films contain QDs and TiO_2_ nanoparticles integrated into the photoresist material. The emission peak at 446 nm, accompanied by a full width at half maximum (FWHM) of 16 nm, corresponds to the emission wavelength of the blue light panel used as the single light emission source. Subsequently, the emission spectra of green QDCC display prominent peaks at a wavelength of 533 nm, accompanied by a FWHM of 22 nm. In a similar manner, it can be observed that red QDCC shows emission peaks at a wavelength of 628 nm, accompanied by a FWHM of 24 nm. The image in the right corner displays a photograph of green QDCC and red QDCC upon being stimulated by a blue light panel.

### 2.4 Experimental measurement setup

**Fig. 6:**
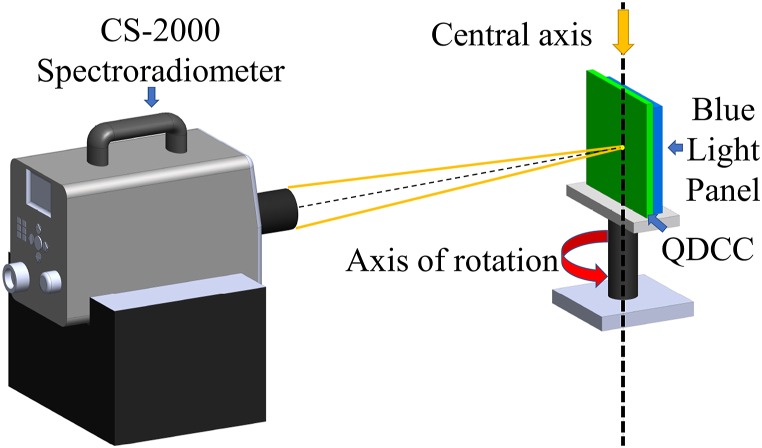
Experimental setup to measure brightness, chromaticity, and spectrum changes at every viewing angle for the Quantum Dot Color Converter (QDCC).

To examine the brightness, chromaticity, and spectrum changes of various QDCCs (QDCC, modified DBR/QDCC, and Y-CF/QDCC) under blue light pumping at each viewing angle, we employ a KONICA MINOLTA CS-2000 spectroradiometer for measurement, as shown in Fig. 6.

**Fig. 7:**
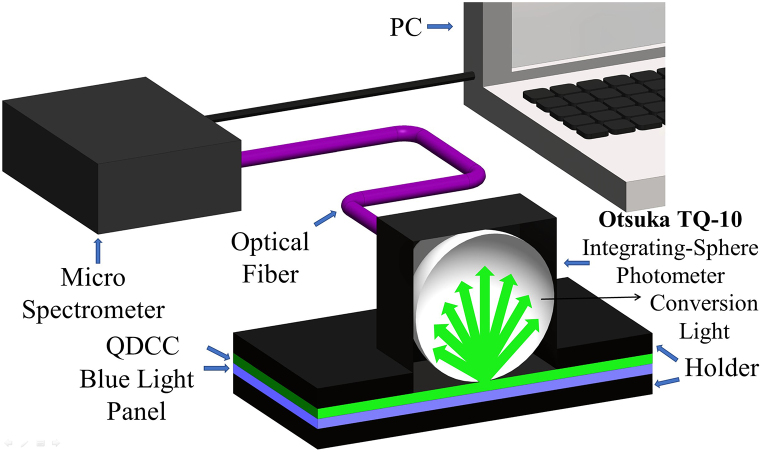
Schematic representation of the integrating sphere (TQ-10) setup for EQE measurements.

Furthermore, for the precise measurement of the external quantum efficiency (EQE) of QDCC, we adopt the TQ-10 system. This measurement technology, developed together by Otsuka Tech Electronics Corp. Ltd. and SynthEdge Advanced Materials Corp. Ltd., is specially designed for micro-LED QDCC analysis. The software also integrates relevant data required for measuring QDCC. Measurement with the TQ-10 system uses vertical sample integration and integrating spheres. QDCC is directly underneath the integrating sphere. This arrangement reduces measurement time and improves user-friendliness compared to typical integrating spheres that need lateral sample and light source placement. Additionally, TQ-10 has a 1.26 cm circular aperture. This allows efficient and precise multi-point sampling, eliminating large-area sampling and enhancing measurement accuracy. The measuring configuration of the integrating sphere device is shown in Fig. 7.

### 2.5 Characterization

**Fig. 8:**
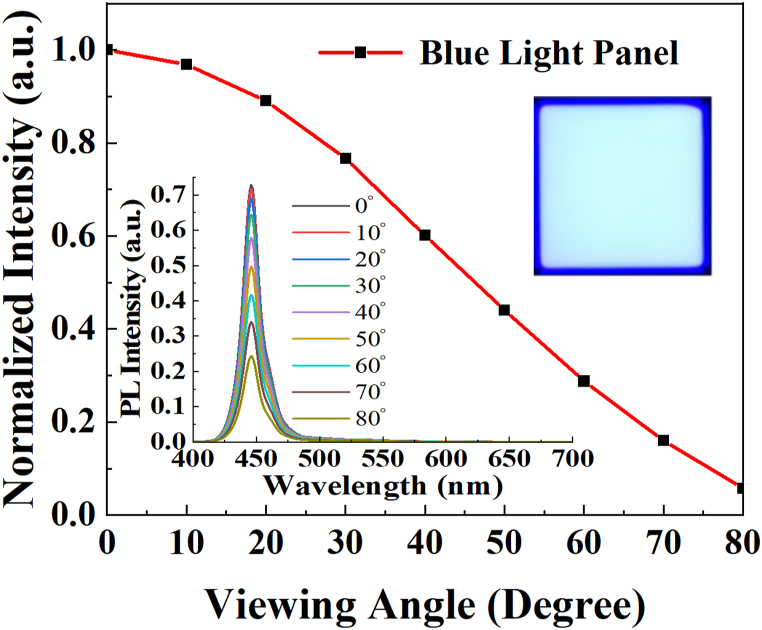
Spatial spectra (inset) and light intensity distribution of the blue light panel‥ (Inset in the upper right: The emission of light from a blue light panel.).

Fig. 8 illustrates the spatial spectra with the viewing angle from 0° to 80° (inset on the left) and the light intensity distribution of the blue light panel. The spatial spectrum and spectral peaks remain unchanged with variations in angles. Furthermore, the distribution of blue light intensity shows a narrower range compared to the Lambertian distribution. (Lambertian distribution: I = I_0_ cosθ, at 60°, I = 0.5 I_0_). The inset in the upper right is the emission of a blue light panel. The blue light panel was purchased from ThousLite, Inc.

## 3 Results and discussion

### 3.1 EQE characterizations

**Fig. 9:**
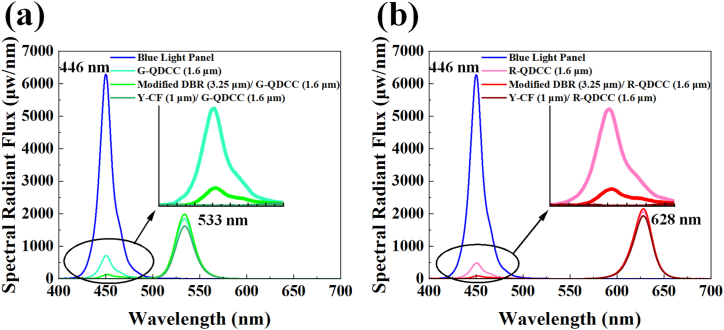
(a) A combined PL spectra of the G-QDCC, modified DBR/G-QDCC, Y-CF/G-QDCC, and a blue light emission by TQ-10 measurement. (b) A combined PL spectra of the R-QDCC, modified DBR/R-QDCC, Y-CF/R-QDCC, and a blue light emission by TQ-10 measurement. Both insets are the magnified plots for the wavelength range of blue leakage emission from QDCCs.

To study the effect of modified DBR and Y-CF on QDCC gamut coverage, QDCCs are measured using an Otsuka TQ-10 integrating sphere system. QDCCs are then alternately placed on a blue light panel emitting at approximately 446 nm, and the resulting emission spectra are captured through an integrating sphere connected to a microspectrometer using one-pass geometry. The EQE of various QDCC structures can be calculated as follows:

$$EQE=\frac{Emitted\;Color\;Converted\;Photons}{Total\;Incident\;Blue\;Photons}=\frac{\int \begin{array}{c} Green\;or\;Red\\ QD\;emission \end{array}\left(\frac{\lambda }{hc}\right)\left[I_{em}^{QD}\left(\lambda \right)-I_{em}^{Ref}\left(\lambda \right)\right]d\lambda }{\int \begin{array}{c} Blue\\ excitation \end{array}\left(\frac{\lambda }{hc}\right)\left[I_{ex}^{Ref}\left(\lambda \right)\right]d\lambda }$$

where QD emission refers to the wavelength range of QD emission, blue excitation is the wavelength range of pump blue light emission, λ is the wavelength, *I*_em_^QD^ and *I*_em_^Ref^ are the spectral response structures at the QD emission wavelength with and without QDCC, respectively. Similarly, I_ex_^Ref^ represents the spectral response at the pump photon wavelength of an individual blue light panel. Thus, the EQE depends on the ratio of converted light intensity to pump blue light intensity. The high EQE value can be interpreted as an indication of the efficient conversion of incident photons in the QDCC structure. Conversely, a low EQE value may suggest significant unconverted photons or the loss of converted photons within the structure. The measured spectra are illustrated in Fig. 9. Fig. 9(a) and (b) show the distinct configurations of green and red QDCC excited by blue light emission. The figure depicts initial blue light emission and QDCC, modified DBR/QDCC, and Y-CF/QDCC, all excited by blue light. It is evident that QDCCs exhibit the most significant blue leakage among the various structures, regardless of whether they are green or red QDCCs. Y-CF/QDCCs almost entirely inhibit blue leakage compared to modified DBR/QDCCs or QDCCs. Although modified DBR/QDCCs display slight blue leakage, it exhibits the highest converted light intensity. Special attention must be paid to the fact that the modified DBR possesses the capability of filtering out blue light emissions. Nevertheless, a slight blue leakage phenomenon in the measurement still exists. That is because the light measured through the integrating sphere encompasses various angles, and the transmittance (or reflectance) of DBR is closely related to the angle at which the light is incident. Therefore, not all angles can maintain identical transmittance (or reflectance). Hence, blue light leakage can still be observed in measurements of the modified DBR/QDCCs.

As previously explained and calculated using the EQE formula, modified DBR can reflect blue light that QDs do not absorb. This enables it to recycle blue light for reuse, resulting in the modified DBR/QDCCs having the most vigorous converted QD emission and, therefore, the highest EQE, regardless of whether green or red QDCCs are used. Although Y-CF suppresses blue leakage at all angles, its R/G band transmittance is lower than that of modified DBR. Consequently, the converted QD emission is the weakest, resulting in the lowest EQE. All EQE data for both green and red QDCCs has been consolidated and shown in Table 1. As can be seen from Table 1, the EQE values of green and red QDCCs are 45.36 % (G-QDCC), 48.66 % (modified DBR/G-QDCC), and 39.97 % (Y-CF/G-QDCC) for green QDCCs. For red QDCCs, the values are 61.78 % (R-QDCC), 64.06 % (modified DBR/R-QDCC), and 57.79 % (Y-CF/R-QDCC), respectively.

**Table 1:**
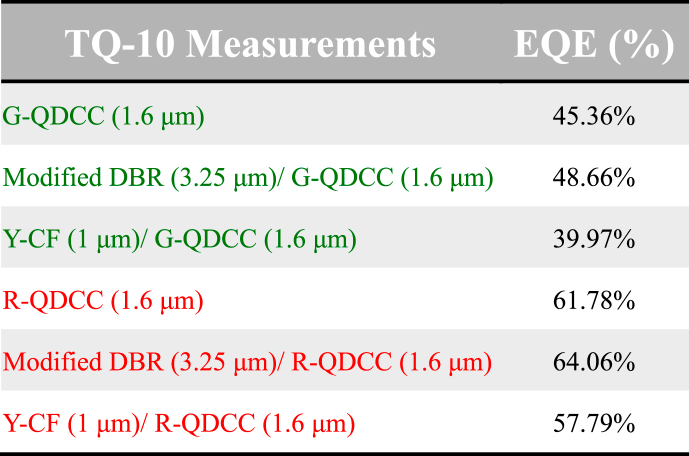
Summary of EQE for green and red QDCC films.

### 3.2 Brightness, chromaticity, and spectral characterization of QDCCs as viewing angle changes

**Fig. 10:**
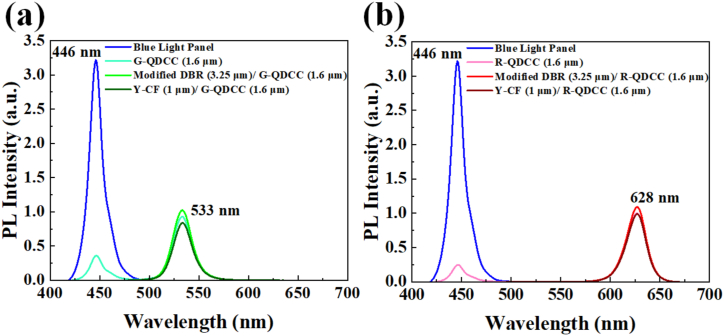
(a) A combined PL spectra of the G-QDCC, modified DBR/G-QDCC, Y-CF/G-QDCC, and a blue light emission by CS-2000 spectroradiometer measurement. (b) A combined PL spectra of the R-QDCC, modified DBR/R-QDCC, Y-CF/R-QDCC, and a blue light emission by CS-2000 spectroradiometer measurement.

However, when green or red QDCCs are measured with a spectroradiometer in the forward direction, the resulting QDCC spectra differ slightly from those obtained with an integrating sphere. In this experiment, a spectroradiometer is employed to measure the light intensity of green and red QDCCs at numerous angles. The alterations in their spectrum and color coordinates are subsequently observed. It is worth noting that a blue light source with a brightness of 2250 nits is used for the measurements in order to comply with the general high ppi (>500 ppi) usage scenario of QDCC-based micro LED displays. Under these conditions, a light-absorbing layer, such as a black matrix (BM) is used around the sub-pixels to minimize crosstalk. Even though the light output is much lower, the QDCC must still withstand the excitation of high-brightness blue light from the front side. If the light conversion efficiency or blue light absorption of the QDCC is low, blue light leakage can occur, resulting in a reduced color gamut. To fully understand the impact of modified DBR/QDCC and Y-CF/QDCC on the color gamut, measurements must be made with a high-intensity blue light source. First, Fig. 10 illustrates the collective PL spectra of QDCC, modified DBR/QDCC, Y-CF/QDCC, and blue light emission. These measurements are conducted in a forward direction. Specifically, the measurement findings of green and red QDCCs are depicted in Fig. 10(a) and (b), respectively.

Obviously, under forward luminance measurement, both modified DBR and Y-CF are able to effectively filter blue leakage, regardless of whether it is green or red QDCCs. Similarly, modified DBR possesses blue light recycling characteristics that are not absorbed by QDs, which results in increased intensity of converted QD emission through the reuse of blue light. Furthermore, QDCCs cannot entirely absorb the excitation of blue light, resulting in their spectrum showcasing the blue leakage phenomenon. In the G/R band, Y-CF's average transmittance in the G/R band is also extremely high, at 94.83 %. Therefore, when converted to QD emission, the intensity of Y-CF/QDCC is only slightly diminished compared to that of QDCC alone.

**Fig. 11:**
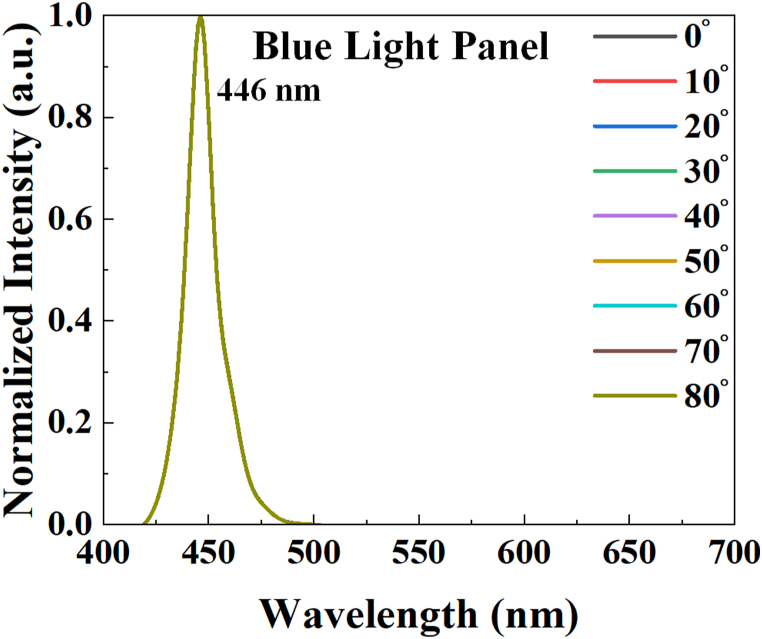
(a) Normalized PL intensity of blue light spectra with a view angle from 0° to 80°. Its forward brightness is 2250 Nits.

Then, we measured and compared the brightness, chromaticity, and spectrum of the emitted light at every viewing angle (0°–80°). First, we analyze the spectrum of the blue light source as it varies with angle. The forward brightness is set at 2250 nits. Very clearly, there is no change in the blue light spectra with respect to angle. The spectra of normalized PL intensity of a blue light panel with a view angle of 0°–80° are shown in Fig. 11.

**Fig. 12:**
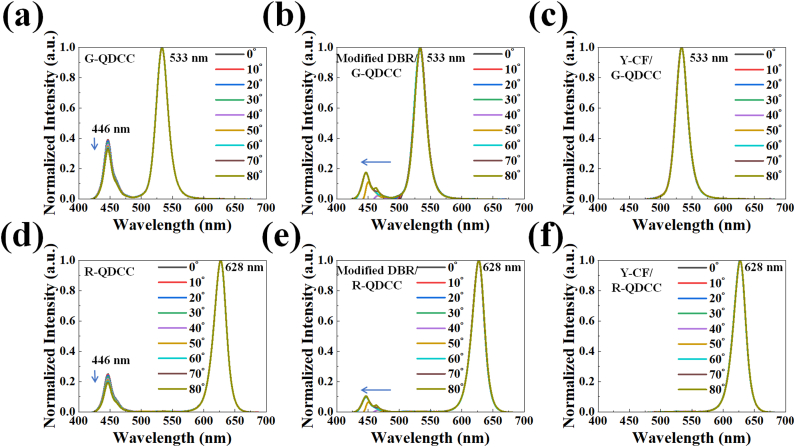
Normalized PL spectra of QDCC, modified DBR/QDCC, and Y-CF/QDCC, respectively; (a), (b), (c) for green, and (d), (e), (f) for red.

Subsequently, we undertake an examination of the spectral attributes shown by green and red QDCCs while the viewing angle undergoes variation. Fig. 12 illustrates the normalization of the PL spectra's characteristic peaks of green and red QDCCs in order to facilitate a comparison of the spectrum variations across different viewing angles. The PL spectra of QD emission from red or green QDCCs remain constant regardless of the viewing angle, which is consistent with the findings from blue light panels. The spectral shape of blue leakage remains largely consistent when observed through the QDCCs, although its intensity does reduce somewhat at higher viewing angles (see Fig. 12; (a) for green QDCC and (d) for red QDCC). Furthermore, it is evident that the PL spectra of the modified DBR/QDCCs exhibit a blue shift in the blue leakage spectrum as the viewing angle increases. Moreover, the magnitude of this blue shift is greater. This phenomenon has been previously elucidated by the fact that the transmittance spectra of DBRs are highly influenced by the angle of incidence. (See Fig. 12; (b) for modified DBR/G-QDCCs and (e) for modified DBR/R-QDCC.) The Y-CF/QDCC structure effectively eliminates blue leakage emissions, leading to the exclusive emission of QD emissions. Furthermore, the PL spectra of the structure exhibit consistent behavior when the viewing angle is altered. This is illustrated in Fig. 12(c) for Y-CF/G-QDCC and (f) for Y-CF/R-QDCC.

**Fig. 13:**
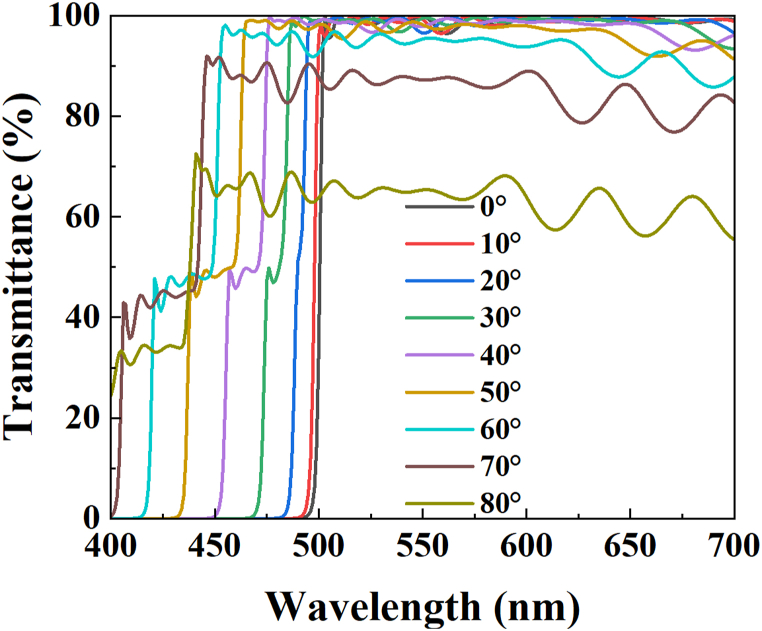
The simulations of the transmittance spectra of modified DBR.

To provide a more comprehensive explanation of the blue leakage phenomenon observed in modified DBR/QDCCs, we employ the Macleod V12.2 optical simulation software to simulate the transmittance spectra of the modified DBR. This simulation allows us to observe how the transmittance spectra vary with changes in the viewing angle, as depicted in Fig. 13. When the viewing angle is forward, the transmittance spectrum of the modified DBR is exactly the same as that of Fig. 2 (blue dashed line). However, as the viewing angle increases, the spectra have been blue-shifted, and the overall transmittance starts to decrease. When viewing angles are greater than 30°, blue light leakage becomes very noticeable. Subsequently, a discernible reduction in the average transmittance is observed when the viewing angles are expanded to 70°. Therefore, we can directly deduce that when QDCC is used with DBR, only the front viewing angle has the best effect of filtering blue leakage and recycling blue light. Hence, it is impossible to maintain the default color purity when the viewing angle is greater than 30°.

Table 2 summarizes the spectroradiometer measurements of QDCC, modified DBR/QDCC, and Y-CF/QDCC with varying angles from 0° to 80°. (a) green QDCCs, and (b) red QDCCs. The measurements include brightness, chromaticity, and peak QD emission data. By referring to Tables 2 and it is evident that the green and red QDCC films of each configuration exhibit very comparable patterns in terms of brightness, chromaticity, and peak QD emission. In general, the three QDCC configurations differ in brightness and chromaticity at different angles. However, the QD emission peak remains unchanged, indicating that the QD emission of QDCC is not affected by the structures of modified DBR and Y-CF. In most cases, the brightness of modified DBR/QDCC is higher than that of QDCC and Y-CF/QDCC, but it starts to decrease dramatically after approaching the viewing angle of 70°, which can be understood from Fig. 13. This is because the modified DBR will reduce the transmittance rapidly after approaching the viewing angle of 70°, resulting in a decrease in emission brightness.

**Table 2:**
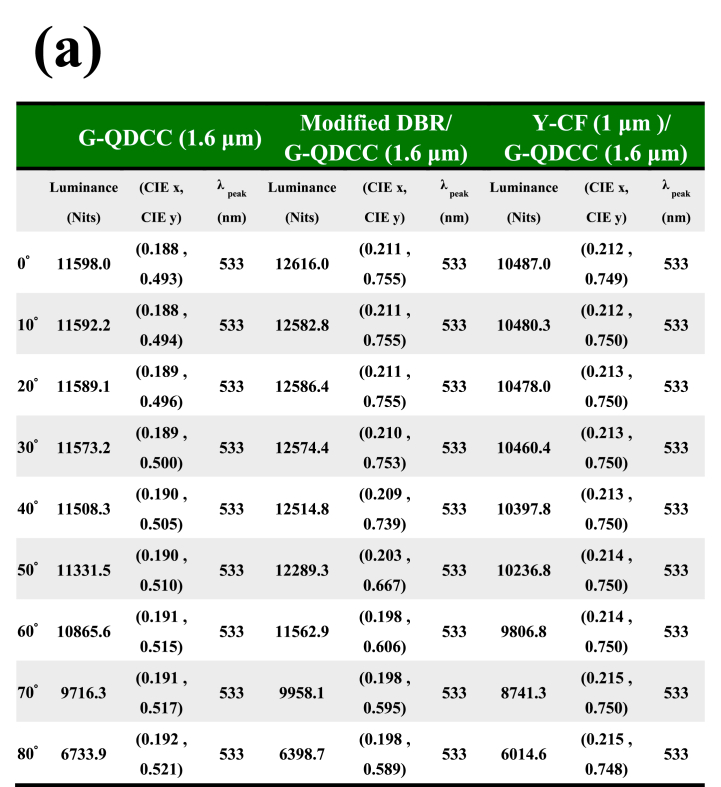

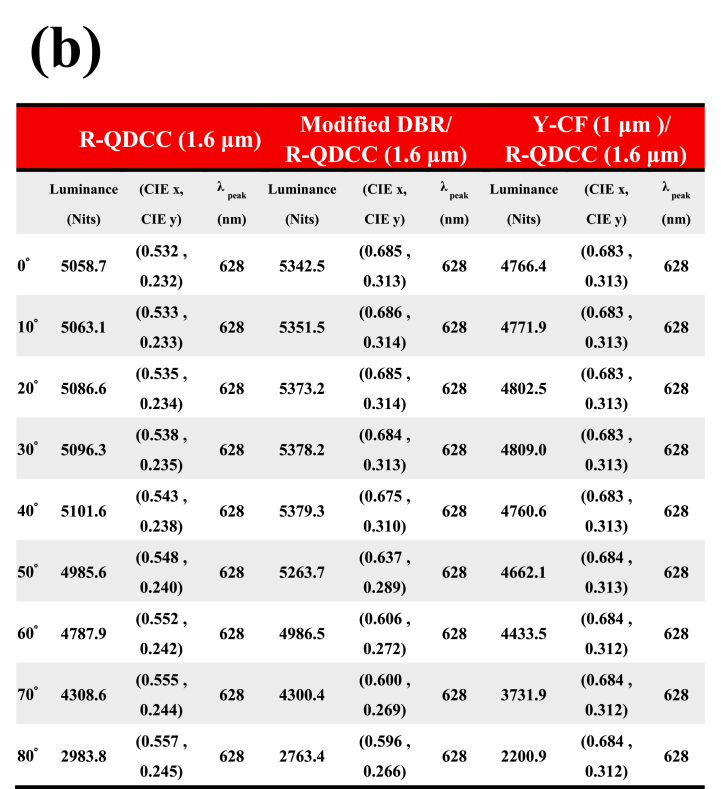
Summary of spectroradiometer measurements performed on QDCC, modified DBR/QDCC, and Y-CF/QDCC with varying angles from 0° to 80° (a) green QDCCs, and (b) red QDCCs. The measurements contain data on luminance, chromaticity, and peak QD emission. (P.S. The chromaticity coordinate of the blue light panel is 0.153, 0.026).

However, regarding chromaticity, G-QDCC has a higher CIE_y_ as the viewing angle increases, while R-QDCC has a higher CIE_x_. This shows that as the viewing angle increases, the color purity also increases. This is because at oblique angles, the longer the path of light through the QDCC, the higher the chance of color conversion. Nevertheless, QDCC itself does not have the effect of suppressing blue leakage, so even though the color purity increases with the angle, the overall color gamut of QDCC is still low due to the blue leakage problem. In addition, in the modified DBR/QDCC case, the blue leakage within 30° is not noticeable yet, and its transparency in the G/R band remains at a high transmittance state of 98.5 %. Thus, its color purity and brightness performance are excellent. However, when the viewing angle exceeds 30°, even though modified DBR/QDCC still has the highest brightness at most angles, its chromaticity decreases rapidly. As a result, the modified DBR/QDCC has the best performance of the three QDCCs in terms of color purity and brightness at viewing angles of less than 30°. Hence, the existing literature on QDCC micro-LED displays extensively investigates the utilization of DBRs in conjunction with QDCCs to improve both brightness and chromaticity. It should be noted that this kind of research is only applicable to displays with a very small viewing angle, such as augmented reality (AR) displays.

It should be emphasized that this is a measurement of a pure QDCC sample. In actual micro-LED display applications, there will be a bank structure to reduce the crosstalk; thus, the variations in viewing angle characteristics will not be exactly the same. Finally, it is found that Y-CF/QDCC shows the most stable chromaticity because Y-CF can completely suppress the blue leakage at all angles and does not affect the spectrum or peak emission of QD emission. However, the price to be paid is a slight reduction in brightness at all viewing angles. This corresponds to the previous results in Fig. 12(c) and (f).

However, it should be noted that the forward incident blue brightness is 2250 Nits. From Table 2, we can see that the forward brightness converted by different types of QDCCs is G-QDCC (11598 Nits), modified DBR/G-QDCC (12616 Nits), Y-CF/G-QDCC (10487 Nits), R-QDCC (5058.7 Nits), modified DBR/R-QDCC (5342.5 Nits), and Y-CF/R-QDCC (4766.4 Nits), respectively. The QDCCs have brightness intensity ratios for G/B and R/B in the following order: 5.2 x, 5.6 x, 4.7 x, 2.2 x, 2.4 x, and 2.1 x. It is obvious that green QDCCs are larger than red QDCCs in the ratio. Despite the lower EQE exhibited by the green QDCC compared to the red QDCC (Table 1), it is essential to consider the visual sensitivity function V(λ) when assessing brightness conversion across different wavelengths. Consequently, green QDCCs have a higher conversion brightness than red QDCCs.

**Fig. 14:**
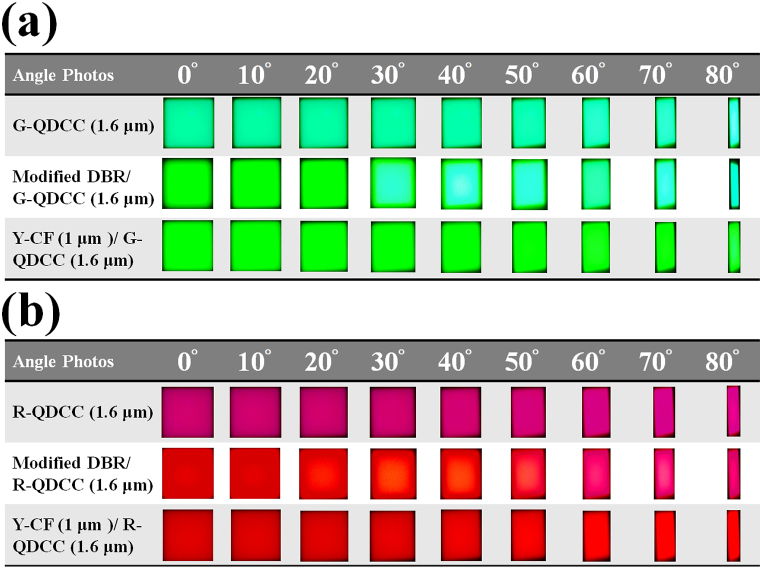
Photographs of QDCC, modified DBR/QDCC, and Y-CF/QDCC with varying angles from 0° to 80° (a) green QDCCs, and (b) red QDCCs.

Fig. 14 shows the photographs of the three structures of QDCCs, modified DBR/QDCCs, and Y-CF/QDCCs in this experiment, with varying angles from 0° to 80° for (a) green QDCCs and (b) red QDCCs. The results are consistent with the experimental results described above for both green QDCCs and red QDCCs. For example: (1). Blue leakage in the QDCC structure leads to poor color purity. (2). After reaching an angle of 30°, the modified DBR/QDCC system exhibits a noticeable phenomenon of color mixing due to blue leakage. Furthermore, as the viewing angle increases, the severity of color mixing becomes more pronounced. (3). The Y-CF/QDCC maintains consistent color across all viewing angles.

**Fig. 15:**
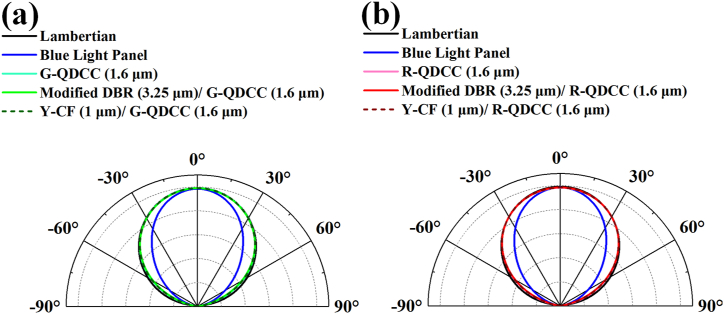
Angular emissive profiles of blue light panel, QDCC, modified DBR/QDCC, and Y-CF/QDCC (a) green QDCCs, and (b) red QDCCs. Black line represents Lambertian emissive profile.

Fig. 15 illustrates the angular-dependent normalized radiant intensity for the blue light panel, QDCC, modified DBR/QDCC, and Y-CF/QDCC, with a black line indicating Lambertian emission for comparison. Fig. 15 (a) displays the angular distribution of green QDCCs, while (b) illustrates the angular distribution of red QDCCs. Nevertheless, like the previous experiments, the green QDCCs and the red QDCCs exhibit concordance. In this experiment, the angular distribution of the green and red QDCCs is very similar to the Lambertian radiation pattern before 50°. However, beyond that angle, the green and red QDCCs’ angular distribution is slightly narrower than the Lambertian radiation pattern. Nevertheless, upon examining the complete angular distribution of QDCC, we can observe that the angular distribution of QDCC is indeed very close to the Lambertian radiation pattern. This phenomenon can be attributed to TiO_2_ nanoparticles as scatterers within the QDCC. When light passes through the QDCC layer, it undergoes significant light scattering, resulting in a broad light distribution. Further analysis reveals that the angle distributions of QDCC and Y-CF/QDCC are almost indistinguishable. The angle distribution of modified DBR/QDCC is slightly smaller than that of QDCC and Y-CF/QDCC. We believe that this change is related to the fact that the transmittance of DBR changes with the viewing angle.

### 3.3 Color gamut

Finally, we organize the chromaticity coordinates in Table 2 into Table 3 to understand the variation of color gamut with angles obtained from the three types of QDCCs in this study. In general research, the brightness and chromaticity measured in the forward direction are used to judge the display performance. In this study, we would like to use the color gamut variation with angle as a more comprehensive way to judge the display performance. Table 3 shows that the modified DBR/QDCC structure has the widest color gamut at the front viewing angle, with an NTSC value of 117.41 % (BT2020 87.67 %). However, as the viewing angle exceeds 30°, the color gamut decreases compared to the Y-CF/QDCC structure at the same viewing angle. The color gamut of the Y-CF/QDCC structure is only slightly lower than that of the modified DBR/QDCC structure, at 115.86 % for NTSC (86.51 % for BT2020). Furthermore, the QDCC structure has a color gamut of only 53.73 % in NTSC and 40.12 % in BT2020, mainly due to blue leakage. In summary, the Y-CF/QDCC structure maintains its color gamut of approximately 116 % NTSC (86.5 % BT2020) at all viewing angles.

**Table 3:**
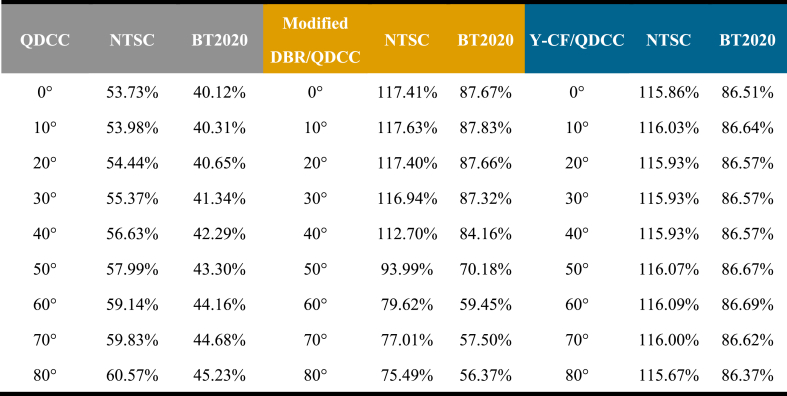
Summary of color gamut performed on QDCC, modified DBR/QDCC, and Y-CF/QDCC with varying angles from 0° to 80°.

We summarize the above results and suggest that Y-CF/QDCC is the best solution for QDCC-based micro-LED displays. It has the most stable color gamut performance that does not change with viewing angle, and the color gamut at the forward viewing angle is only slightly lower than that of the modified DBR/QDCC structure by less than 2 %. In contrast, with the modified DBR/QDCC structure, when the viewing angle exceeds 30°, there is a significant increase in blue light leakage, leading to a rapid reduction in the color gamut. In terms of forward brightness, although Y-CF/QDCC is inferior to the modified DBR/QDCC structure by about 11 % (red) to 17 % (green), we believe that by further developing Y-CF, the performance of the Y-CF/QDCC structure micro-LED display can be further improved.

## 4 Conclusions

In conclusion, we explore and compare the effects of a modified DBR and a Y-CF on the improvement of color saturation, viewing angle, and brightness of a QDCC for micro-LED displays. For a fair comparison, we designed and fabricated a 53-layer high-performance modified DBR that achieves almost complete blue leakage filtering (T %: 0.16 %) and very high transmittance in the G/R band (T %: 96.97 %). In addition, we have prepared a Y-CF that can also effectively filter out blue light (T %: 0.84 %) and has high transmittance in the G/R band (T %: 94.83 %). Measurements show that the modified DBR/QDCC structure has the widest color gamut (117.41 % NTSC) at the forward viewing angle but decreases rapidly after 30° due to the angle dependency effect of DBR. The Y-CF/QDCC structure maintains a stable color gamut (116 % NTSC) at all viewing angles. Overall, the Y-CF/QDCC structure is considered the best solution for current QDCC-based micro-LED displays because it provides the most consistent color performance at all viewing angles with acceptable brightness and an excellent color gamut.

## Data availability

Data will be made available on request.

## CRediT authorship contribution statement

**Bao-Le Dai:** Writing – review & editing, Validation, Investigation. **Jing-Wei Ji:** Visualization, Validation. **Bing-Han Wu:** Visualization, Validation. **Kuan-An Chen:** Validation, Investigation, Conceptualization. **Hideki Kuroda:** Software. **Hung-Chen Kou:** Formal analysis. **Tomohiro Akada:** Software. **Chun-Yu Li:** Writing – review & editing, Writing – original draft, Supervision, Conceptualization.

## Declaration of competing interest

The authors declare that they have no known competing financial interests or personal relationships that could have appeared to influence the work reported in this paper.
