# Supplementary material for: Investigation and comparison of the influence of modified DBR and yellow color filters for quantum dot color conversion-based micro LED applications
Source: Heliyon. 2024 Aug 3;10(16):e35492. doi: 10.1016/j.heliyon.2024.e35492 (PMC11363836; doi:10.1016/j.heliyon.2024.e35492)
Supplement: Multimedia component 2 [file mmc2.docx]

| **Layers** | **Material** | **Evaporation Rate** | **Physical Thickness (nm)** |
| --- | --- | --- | --- |
| **0** | **Glass**  **Substrate** |  |  |
| 1 | Ta_2_O_5_ | 2Å/s | 23.4 |
| 2 | SiO_2_ | 3Å/s | 75.7 |
| 3 | Ta_2_O_5_ | 2Å/s | 55.2 |
| 4 | SiO_2_ | 3Å/s | 75.1 |
| 5 | Ta_2_O_5_ | 2Å/s | 48.8 |
| 6 | SiO_2_ | 3Å/s | 67.2 |
| 7 | Ta_2_O_5_ | 2Å/s | 50.7 |
| 8 | SiO_2_ | 3Å/s | 77.4 |
| 9 | Ta_2_O_5_ | 2Å/s | 51.4 |
| 10 | SiO_2_ | 3Å/s | 73.9 |
| 11 | Ta_2_O_5_ | 2Å/s | 50.0 |
| 12 | SiO_2_ | 3Å/s | 74.8 |
| 13 | Ta_2_O_5_ | 2Å/s | 51.6 |
| 14 | SiO_2_ | 3Å/s | 74.6 |
| 15 | Ta_2_O_5_ | 2Å/s | 51.4 |
| 16 | SiO_2_ | 3Å/s | 74.6 |
| 17 | Ta_2_O_5_ | 2Å/s | 50.3 |
| 18 | SiO_2_ | 3Å/s | 75.6 |
| 19 | Ta_2_O_5_ | 2Å/s | 51.4 |
| 20 | SiO_2_ | 3Å/s | 75.3 |

| **Layers** | **Material** | **Evaporation Rate** | **Physical Thickness (nm)** |
| --- | --- | --- | --- |
| 21 | Ta_2_O_5_ | 2Å/s | 51.2 |
| 22 | SiO_2_ | 3Å/s | 74.5 |
| 23 | Ta_2_O_5_ | 2Å/s | 51.1 |
| 24 | SiO_2_ | 3Å/s | 75.3 |
| 25 | Ta_2_O_5_ | 2Å/s | 50.7 |
| 26 | SiO_2_ | 3Å/s | 75.8 |
| 27 | Ta_2_O_5_ | 2Å/s | 50.9 |
| 28 | SiO_2_ | 3Å/s | 73.7 |
| 29 | Ta_2_O_5_ | 2Å/s | 51.2 |
| 30 | SiO_2_ | 3Å/s | 76.3 |
| 31 | Ta_2_O_5_ | 2Å/s | 51.5 |
| 32 | SiO_2_ | 3Å/s | 74.9 |
| 33 | Ta_2_O_5_ | 2Å/s | 50.6 |
| 34 | SiO_2_ | 3Å/s | 75.3 |
| 35 | Ta_2_O_5_ | 2Å/s | 51.5 |
| 36 | SiO_2_ | 3Å/s | 75.6 |
| 37 | Ta_2_O_5_ | 2Å/s | 50.7 |
| 38 | SiO_2_ | 3Å/s | 74.3 |
| 39 | Ta_2_O_5_ | 2Å/s | 49.8 |
| 40 | SiO_2_ | 3Å/s | 75.3 |
| 41 | Ta_2_O_5_ | 2Å/s | 51.5 |
| 42 | SiO_2_ | 3Å/s | 75.5 |

| **Layers** | **Material** | **Evaporation Rate** | **Physical Thickness (nm)** |
| --- | --- | --- | --- |
| 43 | Ta_2_O_5_ | 2Å/s | 50.3 |
| 44 | SiO_2_ | 3Å/s | 74.2 |
| 45 | Ta_2_O_5_ | 2Å/s | 49.9 |
| 46 | SiO_2_ | 3Å/s | 75.7 |
| 47 | Ta_2_O_5_ | 2Å/s | 50.3 |
| 48 | SiO_2_ | 3Å/s | 71.3 |
| 49 | Ta_2_O_5_ | 2Å/s | 46.5 |
| 50 | SiO_2_ | 3Å/s | 71.0 |
| 51 | Ta_2_O_5_ | 2Å/s | 50.1 |
| 52 | SiO_2_ | 3Å/s | 66.1 |
| 53 | Ta_2_O_5_ | 2Å/s | 24.0 |
|  |  |  |  |
| **Total Time** | 3.61 h | **Actual**  **Time** | ~8 h |
| **λ_0_** | 428 nm |  |  |

**λ_0_** represents the central wavelength during optical design.
